# Taxonomic Diversity of Pico-/Nanoeukaryotes Is Related to Dissolved Oxygen and Productivity, but Functional Composition Is Shaped by Limiting Nutrients in Eutrophic Coastal Oceans

**DOI:** 10.3389/fmicb.2020.601037

**Published:** 2020-12-03

**Authors:** Yaping Wang, Guihao Li, Fei Shi, Jun Dong, Eleni Gentekaki, Songbao Zou, Ping Zhu, Xiaoli Zhang, Jun Gong

**Affiliations:** ^1^School of Marine Sciences, Sun Yat-sen University, Zhuhai, China; ^2^Southern Marine Science and Engineering Guangdong Laboratory (Zhuhai), Zhuhai, China; ^3^Yantai Institute of Coastal Zone Research, Chinese Academy of Sciences, Yantai, China; ^4^School of Science, Mae Fah Luang University, Chiang Rai, Thailand; ^5^School of Life Sciences, Ludong University, Yantai, China; ^6^Guangdong Provincial Key Laboratory of Marine Resources and Coastal Engineering, Guangzhou, China

**Keywords:** functional redundancy, metabarcoding, mixotrophy, nutrient limitation, productivity

## Abstract

Pico-/nanoeukaryotes (P/NEs) comprise both primary producers and bacterial predators, playing important biogeochemical and ecological roles in the marine microbial loop. Besides the difference in size, these small-sized fractions can be distinguished from microplankton by certain functional and ecological traits. Nevertheless, little information is available regarding patterns of their taxonomic and functional diversity and community composition along environmental gradients in coastal marine ecosystems. In this study, we applied high-throughput sequencing of 18S rRNA gene to assess the taxonomic species richness and community composition of P/NEs in surface waters of Bohai Sea and North Yellow Sea, northern China spanning a 600-km distance during summer and winter of 2011. The richness of operational taxonomic units (OTUs) formed a U-shaped relationship with concentration of chlorophyll *a* (Chl-*a*, a proxy of primary productivity), but a stronger, negative relationship with concentration of dissolved oxygen (DO). These two factors also significantly co-varied with the OTU-based community composition of P/NEs. The effect of geographic distance on community composition of P/NEs was negligible. Among the three functional groups defined by trophic traits, heterotrophs had the highest OTU richness, which exhibited a U-shaped relationship with both DO and Chl-*a*. The community of P/NEs was dominated by heterotrophs and mixotrophs in terms of read numbers, which showed a trade-off along the gradient of phosphate, but no significant changes along DO and Chl-*a* gradients, indicating functional redundancy. Similarly, the proportion of phototrophs was significantly and positively correlated with the concentration of silicate. Our results indicate that taxonomic and functional composition of P/NEs are decoupled on a regional scale, and limiting nutrients are important factors in modulating functional composition of these microorganisms in the studied area. These findings contribute toward gaining a better understanding of how diversity of small eukaryotes and their functions are structured in coastal oceans and the effect of environmental changes on the structuring process.

## Introduction

Pico- (0.2–2 μm) and nano-sized (2–20 μm) eukaryotic plankton constitute important components in marine microbial food webs. They frequently comprise major primary producers (Worden and Not, 2008), parasites, symbionts, decomposers (Sherr et al., 2007), and bacterial grazers (Linley et al., 1983; Sherr and Sherr, 1994; Christaki et al., 1999; Massana et al., 2009; Hartmann et al., 2012; Unrein et al., 2014). Due to their small cell size and lack of conspicuous morphological features, pico-/nanoeukaryotes (P/NEs; 0.2–20 μm) are generally difficult to enumerate and identify with high taxonomic resolution using traditional microscopy, especially at the lower hierarchical levels (Moreira and Lopez-Garcia, 2002; Massana, 2011). In the last two decades, application of 18S rRNA gene-based molecular tools has revealed high taxonomic diversity of these small eukaryotes, and picoeukaryotes in particular, in various marine environments, e.g., deep seas (Lopez-Garcia et al., 2001), a bottom euphotic layer of the Pacific Ocean (Moon-van der Staay et al., 2001), a coastal site of English Channel (Romari and Vaulot, 2004), pan-European coastal waters (Massana et al., 2015), and open oceans (de Vargas et al., 2015). Nevertheless, research studies focused on exploring the molecular diversity and biogeography of small marine eukaryotes (Hernandez-Ruiz et al., 2018; Piwosz et al., 2018; Gong et al., 2020), particularly in eutrophic marginal oceans on a large spatial scale are limited.

Concentration of chlorophyll *a* (Chl-*a*) has been widely used as a proxy of phytoplankton biomass and primary productivity, both of which are highly variable across seasons and regions in temperate coastal ecosystems. Picoeukaryotes and nanoeukaryotes are important components in plankton biomass, and often exhibit similar ecological patterns along a productivity gradient. Increase of total Chl-*a* is accompanied by a corresponding increase of the biomass of both pico- and nanophytoplankton. Nevertheless, the relative contributions of these microbial eukaryotes to overall phytoplankton biomass and primary production decline systematically in marine ecosystems (Marañón et al., 2012). This contrasts with microphytoplankton, which contributes an increasing portion of biomass and productivity in more eutrophic waters (Bell and Kalff, 2001; Marañón et al., 2012). Phytoplankton release approximately 20% of their photosynthetic products as dissolved organic carbon (DOC) in surrounding waters in both oligotrophic and eutrophic aquatic habitats (Baines and Pace, 1991; Marañón et al., 2005). The high availability of DOC in eutrophic waters, results in bacterioplankton becoming more productive (Cole et al., 1988; Baines and Pace, 1991), which in turn enhances the abundance and activity of bacterivorus picoeukaryotes and heterotrophic/mixotrophic nanoeukaryotes (Burney et al., 1981; Lonsdale et al., 2006; Šolić et al., 2010). Apart from this bottom-up effect, the abundance of both bacteria and pico-/nanoeukaryotic plankton is controlled in a top-down fashion by microzooplankton (e.g., ciliates), especially under eutrophic conditions (e.g., Šolić et al., 2010; Šimek et al., 2019). Studies that have taken plankton size-fraction into account have shown that a large proportion of oxygen (O_2_) production came from microplankton, whereas pico- and nanoplankton consumed most of the dissolved oxygen (DO) in surface waters of the Canadian Arctic (Harrison, 1986) and a coastal upwelling system (Hernandez-Ruiz et al., 2018). The functional diversity of pico- and nano-sized protists is higher than that of microplankton in coastal oceans (Ramond et al., 2019). Therefore, pico- and nanoplankton are, to some extent, more similar to each other in ecological, physiological and functional aspects than to microplankton, which has prompted us to consider pico- and nanoeukaryotes as a whole in ecological and biogeographic studies. The relationship of overall marine phytoplankton richness with productivity is unimodal (Vallina et al., 2014) and driven by temperature and environmental variability (Righetti et al., 2019). Nevertheless, similar studies on size fractions of plankton have yet to be performed. Furthermore, variability of community composition and structure of P/NEs (including the heterotrophs) in relation to phytoplankton biomass (or productivity) and the main environmental factors driving these changes remain little explored topics.

The Bohai Sea (BHS) and the northern Yellow Sea (NYS) are two shallow and eutrophic coastal basins northwest of the Pacific. These basins are semi-closed, with an average depth of 18 and 40 m, respectively. The recent economic development in the Bohai Economic Circle has resulted in gradually increasing levels of dissolved inorganic nitrogen (DIN), as well as, DIN to phosphate ratio (N:P), thus turning the BHS and NYS into nitrogen-rich and phosphorus-limited basins (Zhao et al., 2016; Yang et al., 2018; Wang et al., 2019). The BHS and NYS ecosystems are variable and mainly controlled by physical processes, including monsoons, tides, and seasonal stratification (Guan, 1994; Wei et al., 2004). The Yellow River directly discharges into the BHS leading to lower salinity and higher nutrient levels in this basin compared to NYS (Chen, 2009). The freshwater discharge causes nutrient replenishment resulting in phytoplankton blooms in spring and August–September. The blooms are accompanied by changes in the concentration of Chl-*a*, which shows distinct seasonality and regional variations (Wei et al., 2004; Liu et al., 2014; Zhou et al., 2017). In general, both Chl-*a* and N:P ratio are higher in the BHS than NYS (Xie et al., 2012). These distinct seasonal and regional attributes of BHS and NYS provide an ideal testing ground for understanding the environmental drivers of the diversity and distribution of P/NEs in coastal oceans that are highly affected by anthropogenic activities. This knowledge is essential for optimizing ecosystem and biogeochemical models (by incorporating or parameterizing pico-/nanoplanktonic components) toward stronger predictive power and subsequent management of such ecosystems.

In this study, we investigated the temporal (summer-winter) and regional variability of diversity and community structure of pico-/nanoeukaryotic plankton in the surface waters of BHS and NYS using high-throughput sequencing of 18S rRNA genes. We hypothesized that: (i) the diversity and community structure of P/NEs would change significantly along the productivity (i.e., Chl-*a*) gradient; (ii) there would be substantial differences in community structure between summer and winter and between the two basins; and (iii) due to higher bioavailability of DOC and primary productivity (Chl-*a*) under highly eutrophic conditions, the pico-/nanoeukaryote community would become functionally more heterotrophic, which would be reflected in increased relative abundance of rRNA gene copies of heterotrophs.

## Materials and Methods

### Sampling and Characterization of Environmental Variables

**FIGURE 1:**
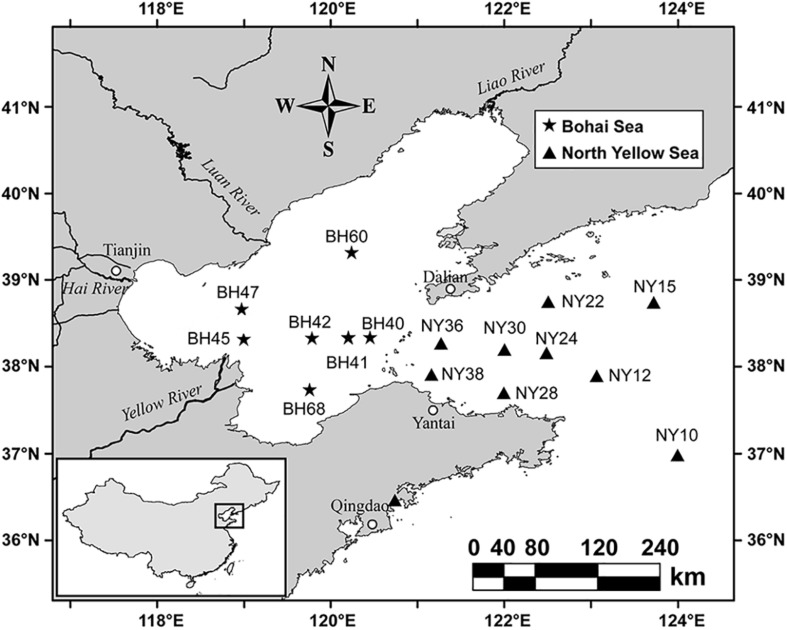
Map of sampling stations at Bohai Sea and North Yellow Sea.

Samples were collected from BHS and NYS during the summer (June 21–28) and winter (November 13–19) cruises of R/V *Dong Fang Hong 2* in 2011 (Figure 1). A total of 28 (16 summertime and 12 wintertime) surface water samples were collected at a depth of 3 m with a rosette of Niskin bottles attached to a conductivity, temperature, depth (CTD) probe frame. At each station, a water sample of 1.5 L was prefiltered through a 20-μm-pore-sized mesh to remove larger plankton and debris, then gently filtered using 0.22-μm-pore-sized polycarbonate membrane filters (47 mm in diameter; Millipore, United States). All membranes bearing the pico- and nano-sized plankton biomass were placed in cryovial tubes and stored in liquid nitrogen for subsequent molecular analyses.

*In situ* measurements of water temperature (Temp), salinity (Sal), depth, and DO concentration were recorded using CTD. Concentration of total chlorophyll *a* (Chl-*a*) was measured on site using an electronic probe (Hydrolab MS5; Hach, United States). At each site, a subsample volume of 100 ml was filtered on board the research vessel through 0.45-μm-pore-sized polyethersulfone membrane filters (25 mm in diameter; Jinteng, Beijing, China) and stored at −20°C for determination of nutrients. The concentrations of nitrate (${\text{NO}}_{3}^{-}$), nitrite (${\text{NO}}_{2}^{-}$), ammonium (${\text{NH}}_{4}^{+}$), dissolved inorganic phosphate (${\text{PO}}_{4}^{3-}$), and silicate (${\text{SiO}}_{3}^{2-}$) in all subsamples were determined with a nutrient AutoAnalyzer (Seal, Germany). Dissolved organic nitrogen (DON) was measured with a TOC-VCPH TOC analyzer (Shimadzu, Japan). The sample ID, sampling sites and dates, as well as, environment variables are supplied in Supplementary Table 1.

### DNA Preparation, Polymerase Chain Reaction (PCR) Amplification, and High-Throughput Sequencing

The FastDNA Spin Kit (MP Biomedical, United States) was used to extract and purify DNA according to the manufacturer’s instructions. The quality of extracted DNA was assessed using gel electrophoresis (1% agarose gels) and quantified using a NanoDrop 2000c spectrophotometer (Thermo Fisher Scientific, United States).

A fragment spanning the V2 and V3 regions of the 18S rRNA gene was amplified using the universal eukaryotic primers 82F (López-García et al., 2003) and 516R (Casamayor et al., 2002). A 10-bp barcode specific to each sample was added to the forward primers. The reaction solutions for PCR were made according to standard conditions for Platinum *Pfx* DNA polymerase (Invitrogen) with 20 ng of environmental DNA as template. Reactions were performed under the following conditions: initial denaturation at 95°C for 2 min; 20 cycles of 95°C for 30 s, 60°C for 30 s, and 72°C for 1 min; and a final extension at 72°C for 7 min. Pyrosequencing was performed with GS-FLX Titanium LV emPCR Kit (Lib-L) on a Roche 454 GS-FLX Titanium sequencer by the BGI Company (Shenzhen, China).

The raw sequencing data (246,066 reads) were processed and analyzed using QIIME (Caporaso et al., 2010) and Mothur v.1.35.1 (Schloss et al., 2009). Quality filtering was performed according to the following criteria: (i) no N’s; (ii) quality score > 25; (iii) no sequencing mismatches within the PCR primer regions; (iv) minimum sequence length of 200 bp and maximum length of 500 bp (excluding PCR primers); and (v) homopolymers ≤ 6. After quality filtering, the wintertime sample NY10W was excluded as only very few reads remained. Putative chimeras were identified with the UCHIME module of USEARCH v.6.0.203 based on the Silva database (release 119) and discarded. The remaining reads were grouped into operational taxonomic units (OTUs) based on a 97% similarity threshold using the UCLUST algorithm. Singletons were excluded from further analysis. Taxonomy annotation was performed against the PR^2^ database (a version based on GenBank v. 230) (Guillou et al., 2012) using UCLUST with default settings. Unassigned reads were discarded. The reads assigned to macroorganisms (e.g., Metazoa, Streptophyta, Rhodophyta, and Ulvophyceae) were treated as contaminants, and hence excluded from subsequent analysis for P/NEs.

To calculate OTU richness, 1590 reads from each sample were randomly re-sampled 10 times. The OTU richness was also partitioned into major groups (e.g., Alveolata, Stramenopiles, Hacrobia, and Opisthokonta), which were subsequently analyzed for spatial and seasonal variability and correlations with environmental variables. For beta diversity analysis, the OTU table was normalized using *edgeR* v. 3.12.1 package (Robinson et al., 2010) in R (R Development Core Team, 2013). This method allows for detection of differentially abundant species as appose to common normalization approaches, such as using simple proportions or rarefying of counts (McMurdie and Holmes, 2014).

The functional structure of P/NEs was assessed using a trait-based approach as previously described (Genitsaris et al., 2015; Ramond et al., 2019). We used the simplified trophic traits (autotrophy, heterotrophy, and mixotrophy) largely because the level of DO, which was mainly driven by photosynthesis and respiration by these trophic groups, was the most important environmental factor co-varying with alpha and beta diversities of P/NEs in this study. The OTU richness and read proportions of these three functional groups were calculated by accumulating those of the affiliated taxonomic groups in a given community (Supplementary Table 2).

### Statistical Analyses

The normality of all variables was tested using Shapiro–Wilk analysis. Student’s *t* tests (for normally distributed variables) and non-parametric Mann–Whitney *U* test (for the variables that showed non-normal distribution) were performed to identify differences in environmental factors, alpha diversity estimators, and the relative proportion of a given taxonomic group between seasons and regions, and among trophic and DO levels. Pearson or Spearman’s correlations between alpha diversity and environmental factors were performed using SPSS v.11.5 (SPSS, Chicago, IL, United States). The statistical differences of the relative proportions of pico-/nanoeukaryotic taxa between the levels of DO and Chl-*a* were assessed by one-way ANOVA and least significant difference (LSD) *post hoc* test. Visualization of community relatedness was conducted using non-metric multiple dimensional scaling (NMDS), which was based on Bray-Curtis similarity matrices. Redundancy analysis (RDA) was used to explore the co-variations between environmental parameters and community structure of P/NEs. Only the variables (i.e., Chl-*a* and DO) that were statistically significant based on forward selection were plotted. Analysis of Similarity (ANOSIM) was executed to test hypotheses regarding variation of community structure of P/NEs between seasons, regions, or among different levels of environmental variables. All analyses of community structure were carried out using *vegan* in R (v. 2.4-3) (Oksanen et al., 2013).

## Results

### Spatial and Seasonal Variations of Environmental Factors

Most of the environmental variables determined for the 28 surface water samples (i.e., temperature, DO, pH, Chl-*a*, ${\text{NO}}_{3}^{-}$, ${\text{NO}}_{2}^{-}$, ${\text{SiO}}_{3}^{2-}$, TN, DON, and P:Si) demonstrated distinct seasonality (Supplementary Table 1). The water temperature ranged from 13.15 to 21.47°C in summer and from 12.09 to 13.67°C in winter across BHS and NYS (*P* < 0.001). The level of Chl-*a* was significantly higher in summer (mean ± SE, 3.34 ± 0.32 μg L^–1^) than in winter (0.27 ± 0.03 μg L^–1^; *P* < 0.001), and so was the concentration of DO (7.41 ± 0.17 vs. 3.95 ± 0.04 mg L^–1^, *P* < 0.001). The concentrations of ${\text{NO}}_{3}^{-}$ (1.20 ± 0.16 vs. 2.49 ± 0.23 μM, *P* < 0.001), ${\text{NO}}_{2}^{-}$ (0.25 ± 0.02 vs. 0.52 ± 0.06 μM, *P* < 0.001), DON (9.52 ± 1.03 vs. 13.56 ± 1.71 μM, *P* = 0.050), and ${\text{SiO}}_{3}^{2-}$ (1.10 ± 0.16 vs. 4.30 ± 0.63 μM, *P* < 0.001) were relative lower in the summertime samples, due to highly abundant phytoplankton standing stock in summer and increased nutrient uptake. ${\text{NH}}_{4}^{+}$ was the dominant DIN species (78%). The N:P ratios varied greatly across all samples, ranging from 19.7 to 2170, with relatively higher values in winter (440 ± 175) than in summer (138 ± 20). The N:Si ratio ranged from 0.7 to 50, but was not significantly different between these two seasons (14.7 ± 3.6 vs. 8.8 ± 3.7, *P* = 0.070). The concentrations of Chl-*a*, DO, pH, ${\text{NO}}_{3}^{-}$, ${\text{NO}}_{2}^{-}$, DON, and ${\text{SiO}}_{3}^{2-}$ were higher in the BHS, while salinity, ${\text{NH}}_{4}^{+}$, ${\text{PO}}_{4}^{3-}$, N:P, and N:Si tended to be higher in the NYS, but these differences were not significant.

### Variations in Richness of Pico-/Nanoeukaryotes

**FIGURE 2:**
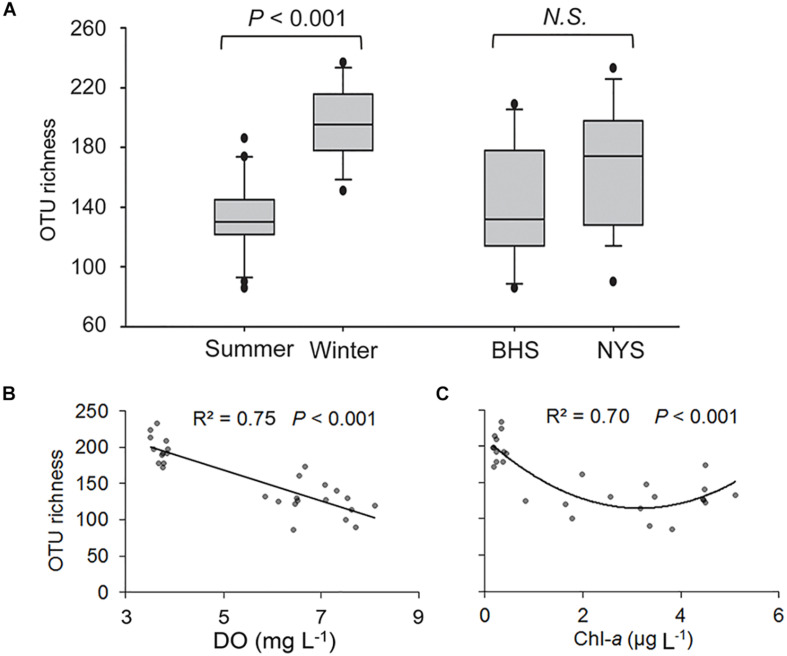
Variations in OTU richness of pico-/nanoeukaryotes in surface waters of Bohai Sea (BHS) and North Yellow Sea (NYS). **(A)** Box plots showing the OTU richness was significantly higher in winter than in summer across two basins, but not between BHS and NYS. **(B,C)** Regression analyses showing the relationships between OTU richness and two of the most important factors, dissolved oxygen (DO, **B**) and chlorophyll *a* (Chl-*a*, **C**). *N.S.*, no significant differences (*P* > 0.05).

After quality filtering, a total of 94,642 reads and 893 OTUs were retained for the 28 surface samples, among which 417 OTUs were detected in both summer and winter samples, and 611 OTUs in both basins (Supplementary Table 3). The OTU richness of P/NEs was significantly higher in the winter (198 ± 5) than in summer (131 ± 7) across the whole area studied (*P* < 0.001; Figure 2A). The P/NEs in NYS (167 ± 10) appeared to be more diverse than in BHS (143 ± 12), however, this basin-wise difference was not statistically supported (*P* = 0.147, Figure 2A).

Both linear and non-linear regression analyses were performed to explore the relationships between OTU richness of P/NEs and the abiotic or biotic factors, among which DO and Chl-*a* had the highest coefficients of determination. The OTU richness linearly decreased with DO, which explained 75% of observed variance (*P* < 0.001; Figure 2B), whereas a lower variance (70%) was explained by Chl-*a* in a quadratic fitting (*P* < 0.001; Figure 2C). In particular, the relationship between OTU richness of P/NEs and Chl-*a* was U-shaped, i.e., richness decreased and then increased along the Chl-*a* gradient, reaching the minimum at an intermediate level (∼ 3 μg Chl-*a* L^–1^) of phytoplankton biomass (Figure 2C).

**FIGURE 3:**
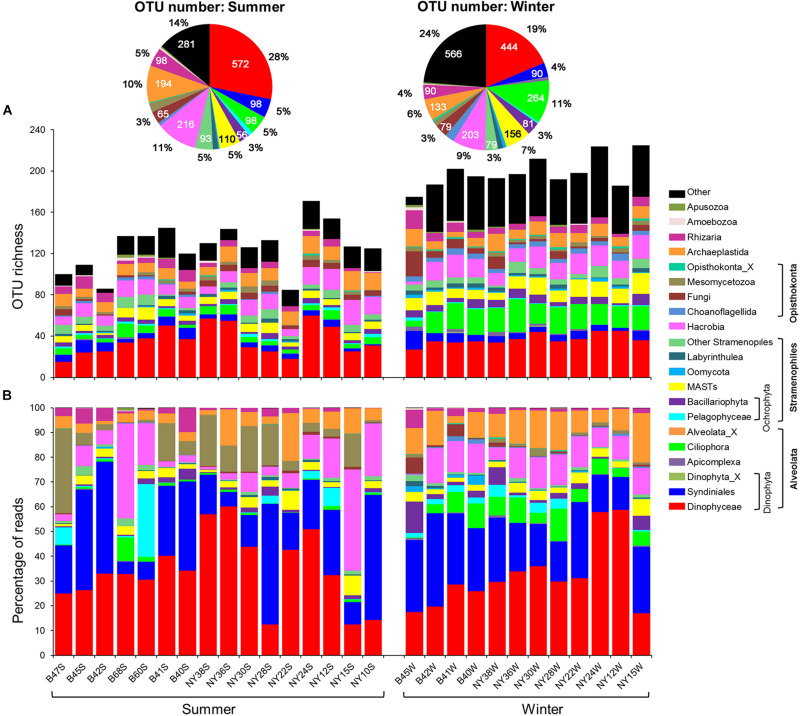
Variations in OTU richness and community composition of pico-/nanoeukaryotes. **(A)** The column chart shows the variation in OTU numbers of major taxa among samples; the pie charts summarize the OTU numbers and proportions of major taxa in summer and winter, respectively. **(B)** Relative proportions of pyrotags of major taxa across samples. MASTs, marine stramenopiles.

A quarter of pico-/nanoeukaryote OTUs were affiliated with Dinophyceae, of which the percentage of OTU number varied greatly (15 ∼ 44%) among samples. The OTU numbers of Hacrobia (on average 10%), Ciliophora (7%), Archaeplastida (8%), marine stramenopiles (MASTs, 6%), and Syndiniales (5%) were also abundant. Significant summer-winter differences in percentage of OTU number were observed for Dinophyceae (28 vs. 19%), Hacrobia (11 vs. 9%), Archaeplastida (10 vs. 6%), Syndiniales (5 vs. 4%), and Rhizaria (5 vs. 4%), whereas the opposite was true for Ciliophora (5 vs. 11%) and MASTs (5 vs. 7%; Figure 3A). The OTU richness of Syndiniales, Ciliophora, MASTs, Bacillariophyta, Cryptophyta, Choanoflagellida, and Apicomplexa formed U-shaped relationships with both DO and Chl-*a*, with the exception of Cryptophyta and Apicomplexa, which formed a negative relationship with DO (0.29 ≤ *R*^2^ ≤ 0.80, *P* ≤ 0.004; Supplementary Figures 1, 2).

### Community Structure, Seasonal, and Regional Variations

Overall, the reads of pico-/nanoeukaryotes were dominated by Alveolata (61.1%), which was comprised of Dinophyceae (33.4%), Syndiniales (24.0%), Ciliophora (3.6%), Apicomplexa (0.1%), and Perkinsida (0.01%; Figure 3B). Hacrobia (11.0%; mostly Haptophyta and Cryptophyta), Stramenopiles (10.1%), Chlorophyta (8.3%), Opisthokonta (7.2%), and Rhizaria (2.2%) were also present. Most of Stramenopiles was affiliated with MASTs (3.3%), Pelagophyceae (2.7%), Bacillariophyta (2.2%), Labyrinthulea (0.4%), Oomycota (0.4%), Chrysophyceae-Synurophyceae (0.2%), and Bicoecea (0.2%). The reads of marine Ochrophyta (MOCH), *Pirsonia*, Dictyochophyceae, Bolidophyceae-and-relatives, Eustigmatophyceae, and Raphidophyceae were each less than 0.1%. Over 50% of reads of Chlorophyta belonged to Mamiellophyceae. The read percentages of other higher-ranking taxa, such as Apusozoa and Amoebozoa, were on average less than 1% (Figure 3B).

**FIGURE 4:**
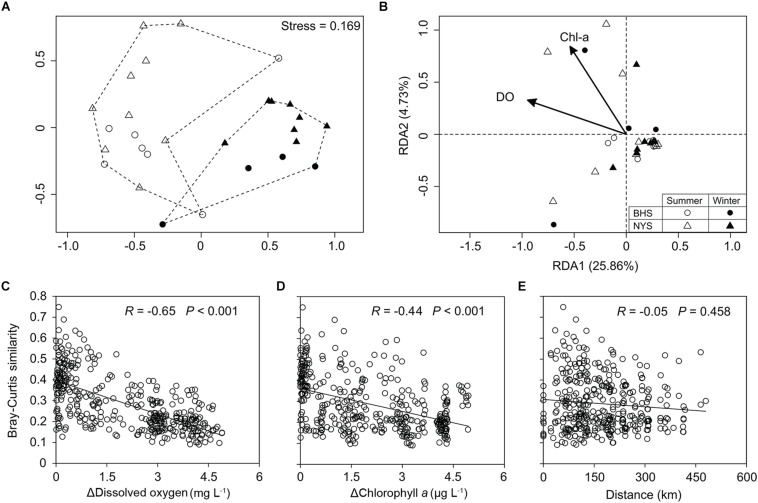
Variations in taxonomic community composition of pico-/nanoeukaryotes. **(A)** A non-metric multidimensional scaling plot depicting the distinct seasonality of taxonomic community structure in NYS and BHS. **(B)** Plot of redundancy analysis (RDA) showing dissolved oxygen and chlorophyll *a* significantly (Monte Carlo test, *P* < 0.05) co-varied with the taxonomic community composition. **(C–E)** Scatter plots showing that the similarity in taxonomic community composition was significantly decreased with the differences in dissolved oxygen **(C)** and chlorophyll *a* **(D)**, but not with that of geographic distance between samples **(D)**.

The NMDS plot showed that the community structure of P/NEs in the summertime samples of both basins was more divergent and well separated from the wintertime samples (Figure 4A). The community structure of P/NEs was significantly different between summer and winter (ANOSIM, *R* = 0.698, *P* = 0.001, Table 1), whereas this was not the case between BHS and NYS (*R* = 0.023, *P* = 0.278, Table 1). The RDA plot revealed that the community structure of P/NEs co-varied significantly with DO and Chl-*a* (*P* < 0.05; Figure 4B). Correlations between paired community Bray-Curtis similarity and pairwise differences in DO and Chl-*a* also showed a stronger effect of the former (*R* = −0.65, *P* < 0.001) than the latter (*R* = −0.44, *P* < 0.001; Figures 4C,D). The effect of geographic distance on the taxonomic community composition of P/NEs was not significant (*R* = −0.05, *P* = 0.46; Figure 4E).

**TABLE 1:** ANOSIM testing the differences in taxonomic community structure of P/NEs between seasons (summer and winter), basins (BHS and NYS), and among levels of dissolved oxygen (DO) and Chl-*a*.

	*R*	*P*
Season	0.698	**0.001**
Basin	0.023	0.278
DO (Global test)	0.806	**0.001**
3–5 vs. 5–7 mg L^–1^	0.860	**0.001**
3–5 vs. 7–9 mg L^–1^	0.931	**0.001**
5–7 vs. 7–9 mg L^–1^	0.324	**0.001**
Chl-*a* (Global test)	0.662	**0.001**
0–1 vs. 1–4 μg L^–1^	0.828	**0.001**
0–1 vs. 4–6 μg L^–1^	0.676	**0.001**
1–4 vs. 4–6 μg L^–1^	0.069	0.265

*Significant P-values (≤0.05) are highlighted in bold.*

**FIGURE 5:**
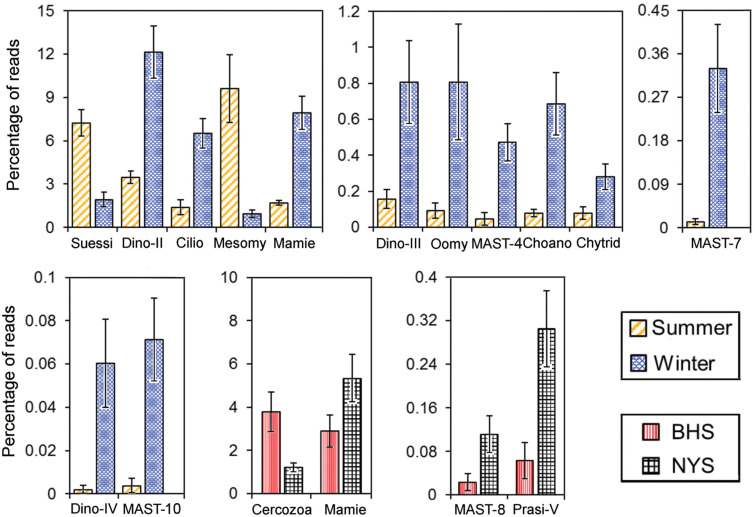
The pico-/nanoeukaryotic taxa of which the relative proportions were significantly different (*P* ≤ 0.05 by Student’s *t*-test or Mann–Whitney *U* test) between summer and winter, or between BHS and NYS. The error bars indicate standard errors. Asco, Ascomycota; Basidio, Basidiomycota; Choano, Choanoflagellida; Chytrid, Chytridiomycota; Cilio, Ciliophora; Crypto, Cryptophyta; Dino-II, Dino-Group-II; Dino-III, Dino-Group-III; Dino-IV, Dino-Group-IV; Labyri, Labyrinthulea; Mamie, Mamiellaceae; MASTs, marine stramenopiles; Mesomy, Mesomycetozoa; Oomy, Oomycota; Prasi-V, Prasino-Clade-V; Prym, Prymnesiales; Suessi, Suessiales.

Comparisons between summer and winter revealed that the read percentages of many groups showed a distinct seasonality (Figure 5 and Supplementary Table 4). The proportions of Suessiales (7.2 ± 0.92 vs. 1.9 ± 0.50%) and Mesomycetozoa (9.6 ± 2.36 vs. 0.9 ± 0.25%) were much higher in the summer than in the winter (*P* < 0.001). In contrast, Dino-Group-II (3.4 ± 0.44 vs. 12.1 ± 1.82%), Dino-Group-III (0.2 ± 0.05 vs. 0.8 ± 0.23%), Dino-Group-IV (0 vs. 0.1 ± 0.02%), Ciliophora (1.4 ± 0.55 vs. 6.5 ± 1.02%), Mamiellophyceae (1.7 ± 0.21 vs. 8.0 ± 1.13%), Chytridiomycota (0.1 ± 0.03 vs. 0.3 ± 0.07%), Oomycota (0.1 ± 0.04 vs. 0.8 ± 0.32%), MAST-4 (0.0 ± 0.03 vs. 0.5 ± 0.10%), and MAST-7 (0.0 ± 0.01 vs. 0.3 ± 0.09%) were significantly more abundant in the winter (*P* < 0.05). Dino-Group-I was the most abundant among the four Dino-groups, accounting for approximately 20.5 and 10.8% of pico-/nanoeukaryotic reads in summer and winter, respectively, but this seasonal difference was not significant (*P* = 0.19; Supplementary Table 4).

Despite the community structures of P/NEs not being significantly different between BHS and NYS, basin-wise differences in relative proportion were detected for several lineages (Figure 5 and Supplementary Table 4). These included Mamiellophyceae (2.9 ± 0.76 vs. 5.3 ± 1.12%), MAST-8 (0.0 ± 0.02 vs. 0.1 ± 0.03%), Prasino-Clade-V (0.1 ± 0.03 vs. 0.3 ± 0.07%), and Cercozoa (3.8 ± 0.92 vs. 1.2 ± 0.21%) (Figure 5 and Supplementary Table 4).

### Changes in Taxonomic Composition Along Dissolved Oxygen Gradient and Productivity

**FIGURE 6:**
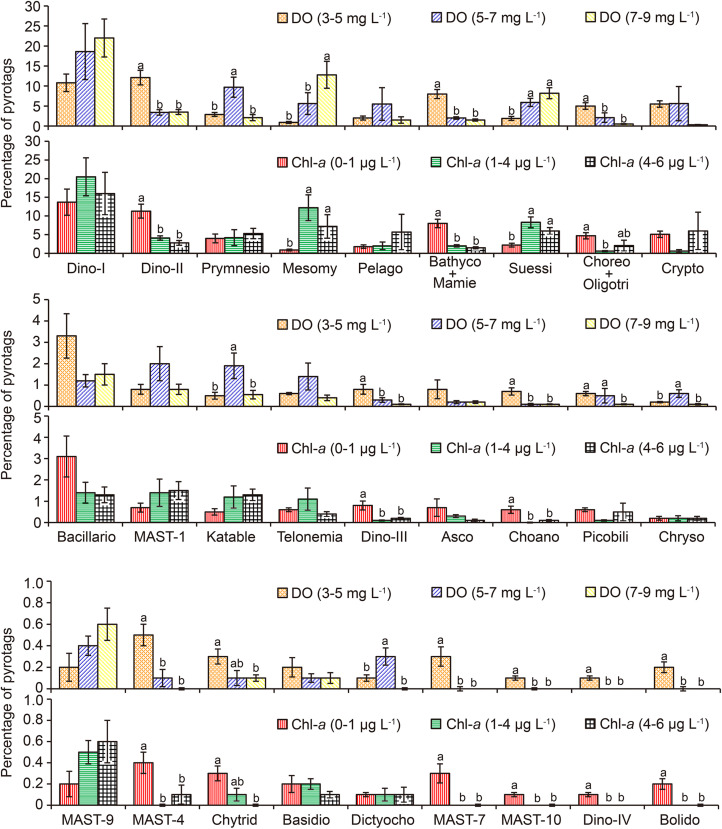
Comparisons of the relative proportions of pico-/nanoeukaryotic taxa between the levels of dissolved oxygen (DO) and chlorophyll *a* (Chl-*a*). The error bars indicate standard errors. Different lowercase letters indicate significant differences (*P* ≤ 0.05 using Least-Significant Difference test) in percentage of pyrotags of a taxon between two levels of DO or Chl-*a.* Absence of letters indicates insignificant effect (*P* > 0.05 by one-way ANOVA) of DO or Chl-*a* on the relative proportion of a taxon. Asco, Ascomycota; Bacillario, Bacillariophyta; Basidio, Basidiomycota; Bathyco + Mamie, Bathycoccaceae + Mamiellaceae; Bolido, Bolidophyceae; Choano, Choanoflagellida; Choreo + Oligotri, Choreotrichia + Oligotrichia; Chryso, Chrysophyceae-Synurophyceae; Chytrid, Chytridiomycota; Crypto, Cryptophyta; Dictyocho, Dictyochophyceae; Dino-I, Dino-Group-I; Dino-II, Dino-Group-II; Dino-III, Dino-Group-III; Dino-IV, Dino-Group-IV; Katable, Katablepharidophyta; MASTs, marine stramenopiles; Mesomy, Mesomycetozoa; Pelago, Pelagophyceae; Picobili, Picobiliphyta; Prymnesio, Prymnesiophyceae; Suessi, Suessiales.

In order to explore how these two factors affected community structure, the relative proportions of major lineages were statistically compared among three levels of DO (low: 3–5 mg L^–1^; intermediate: 5–7 mg L^–1^; and high: 7–9 mg L^–1^) and Chl-*a* (low: 0–1 μg L^–1^; intermediate: 1–4 μg L^–1^; and high: 4–6 μg L^–1^; Figure 6 and Supplementary Table 5). Along the DO gradient, 11 taxonomic groups had the highest pyrotag proportions under low DO conditions. These included Dino-Group-II (mean 12.1, 3.4, and 3.5%); Bathycoccaceae + Mamiellaceae (7.9, 1.8, and 1.2%), Choreotrichia + Oligotrichia (5, 2.1, and 0.5%), Dino-Group-III (0.8, 0.3, and 0.1%); Choanoflagellida (0.7, 0.1, and 0.1%), MAST-4 (0.5%, 0.1%, and 0); Chytridiomycota (0.3, 0.1, and 0.1%); MAST-7 (0.3%, 0, and 0); MAST-10 (0.1%, 0, and 0); Dino-Group-IV (0.1%, 0, and 0); and Bolidophyceae (0.2%, 0, and 0; Figure 6). The pyrotag proportions of these taxa were the highest at the lowest level of Chl-*a* as well (Figure 6).

In contrast, among the three levels of DO, Prymnesiophyceae (2.9, 9.7, and 2.1%), Katablepharidophyta (0.5, 1.9, and 0.6%), Chrysophyceae-Synurophyceae (0.2, 0.6, and 0.1%), and Dictyochophyceae (0.1%, 0.3%, and 0) exhibited significantly higher proportions at intermediate DO (*P* < 0.05). However, the pyrotag proportions of these taxa were not significantly different among any of the three Chl-*a* levels (*P* > 0.05; Figure 6). Finally, only pyrotags of Mesomycetozoa (0.9, 5.6, and 12.8%) and Suessiales (1.9, 5.9, and 8.2%; Figure 6) peaked under high DO conditions and at intermediate levels of Chl-*a*.

### Variations in Richness of Functional Groups and in Community Functional Structure

**FIGURE 7:**
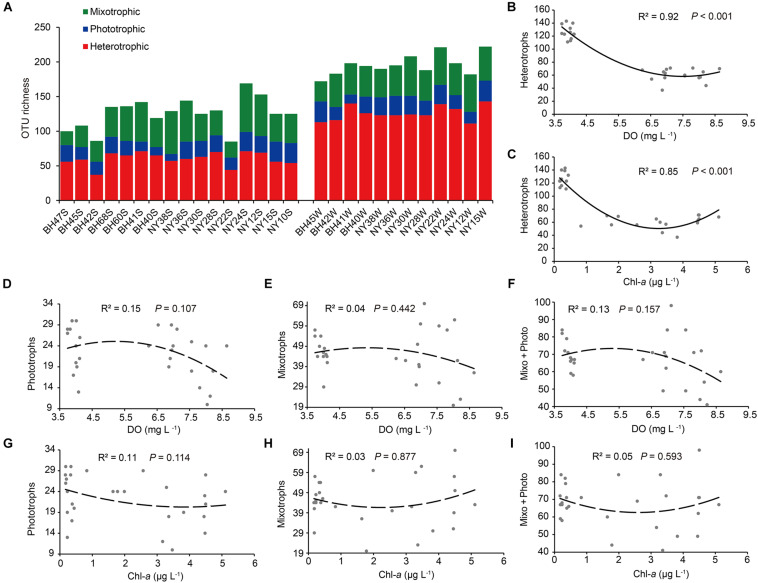
**(A)** The OTU richness of heterotrophs, phototrophs, and mixotrophs. **(B–I)** Variations in OTU numbers of these functional groups in relation to dissolved oxygen (DO) and chlorophyll *a* (Chl-*a*) concentrations. Note that the relationships between OTU richness of heterotrophs and DO and Chl-*a* are highly significant and U-shaped. The abbreviation “Mixo + Photo” refers to pigmented pico-/nanoeukaryotes.

The OTU richness of heterotrophs ranged from 37 to 143, ranging from 41.5 to 69.3% across all samples. In contrast, the OTU richness of mixotrophs (20 ∼ 70) and phototrophs (10 ∼ 30) was much lower and with narrower ranges in the pico-/nanoeukaryote communities (Figure 7A). Regarding seasonal and basin-wise comparisons, a significant difference in OTU richness was only found for the heterotrophs between winter (126 ± 3.0) and summer (60 ± 2.4; *P* < 0.001). Among all determined environmental variables, DO (*R*^2^ = 0.92, *P* < 0.001) and Chl-*a* (*R*^2^ = 0.85, *P* < 0.001) were the most significant, consistently showing a U-shaped relationship with heterotrophic OTU richness (Figures 7B,C). The richness of phototrophs, mixotrophs and mixotrophs + phototrophs (i.e., pico-/nanophytoplankton) tended to be maximized at the intermediate levels of DO, while richness was minimized at the intermediate Chl-*a* concentrations; nevertheless, these quadratic relations were weak and not statistically supported (*R*^2^ < 0.2, *P* > 0.05; Figures 7D–I).

**FIGURE 8:**
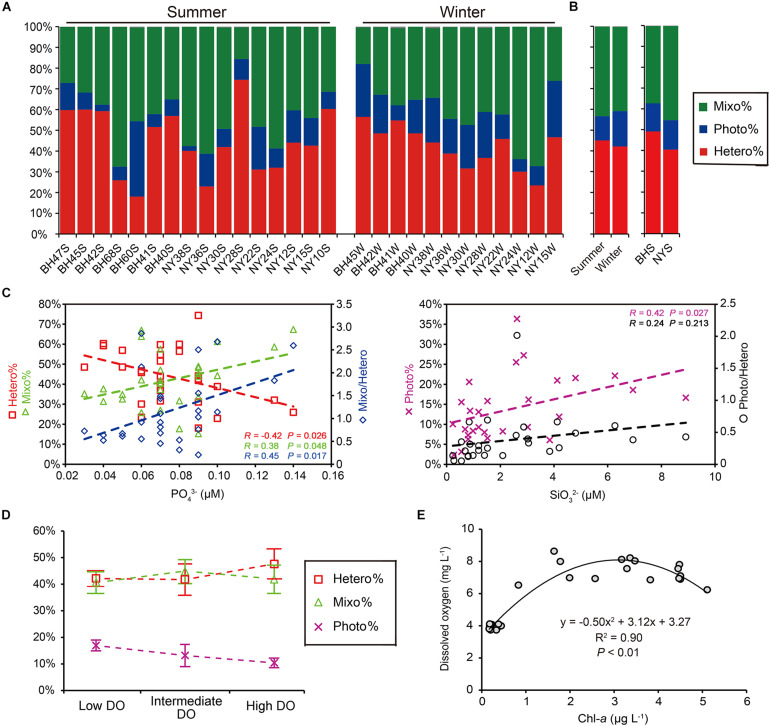
Functional composition of pico-/nanoeukaryotic community as shown by the pyrotag proportions of heterotrophs (Hetero%), phototrophs (Photo%), and Mixotrophs (Mixo%) in the communities. **(A)** Variation among individual samples. **(B)** Comparisons between two seasons and between basins, none of which was significant (*P* > 0.05). **(C)** The Hetero% was negatively correlated with concentration of phosphate, and Mixo% was positively correlated with concentration of phosphate, resulting in a higher Mixo/Hetero ratio at a higher level of phosphate (*P* < 0.05). Both Photo% was positively related to concentration of silicate (*P* < 0.05), whereas the ratio of phototrophs to heterotrophs (Photo/Hetero) was not. **(D)** There were no significantly differences in percentages of pyrotags (mean and standard error) of three trophic groups across the low, intermediate and high levels of dissolved oxygen (DO). **(E)** The humped relationship between concentrations of DO and chlorophyll-*a* observed in the present study.

The read proportions of the three ecological groups in the pico-/nanoeukaryotic community varied greatly across the two seasons and regions (Figure 8A). The percentages of heterotrophs (Hetero%) and mixotrophs (Mixo%) in the community varied greatly, accounting for 18∼74% and 15∼67% among all samples, respectively. The contribution of phototrophs (Photo%) was consistently low (2∼36%). The pyrotag proportions of heterotrophs and mixotrophs appeared to be higher in the summer, whereas phototrophs became more abundant in the winter; however, these differences were not statistically supported (*P* > 0.05; Figure 8B). Similarly, the proportions of these three functional groups were not significantly different between BHS and NYS either (*P* > 0.05). Correlations between the proportions of functional groups and all determined environmental variables showed contrasting patterns: the Hetero% was significantly and negatively correlated with ${\text{PO}}_{4}^{3-}$ (*R* = −0.42, *P* = 0.026), whereas both the Mixo% (*R* = 0.38) and the ratio of mixotrophs to heterotrophs (Mixo/Hetero; *R* = 0.45) were significantly and positively correlated with ${\text{PO}}_{4}^{3-}$ (*P* < 0.05) (Figure 8C). The Photo% increased linearly with the concentration of ${\text{SiO}}_{3}^{2-}$ (*R* = 0.42, *P* = 0.027). Nevertheless, the correlation between the ratio of phototrophs to heterotrophs (Photo/Hetero) and ${\text{SiO}}_{3}^{2-}$ was weak and insignificant (*R* = 0.24, *P* > 0.05; Figure 8C).

The changes in functional groups across the three DO levels showed some general trends. Hetero% appeared to be the highest (47.6%) in the highly oxygenated waters; Photo% was highest (17.0%) at low DO level, but progressively decreased at intermediate (10.4%) and high DO levels; and Mixo% peaked (50%) at the intermediate DO level (Figure 8D). Nevertheless, the statistical comparisons among the three levels were not significant (*P* > 0.05). Across the gradient of phytoplankton biomass, the functional structure of P/NEs was not discernible either (data not shown), despite there being a high and positive correlation between DO and Chl-*a* (*R* = 0.78, *P* < 0.001). This was probably due to the fact that in the present study the increase of DO with Chl-*a* was not monotonic and could fit better with a parabolic curve instead (*R*^2^ = 0.90, *P* < 0.01; Figure 8E): when Chl-*a* was relatively lower (<3 μg L^–1^), the DO in surface waters increased along with Chl-*a*; whereas the slope became negative when Chl-*a* was beyond the turning point (>3 μg L^–1^; Figure 8E).

## Discussion

The nutrient levels in the surface waters of BHS and NYS determined in this study showed that the DIN was dominated by high concentration of ${\text{NH}}_{4}^{+}$ in summer and by ${\text{NO}}_{3}^{-}$ in winter; the on average concentration of ${\text{PO}}_{4}^{3-}$ was very low (0.068 μM); and N:P ratio (on average 355) was much higher than the Redfield ratio (16:1). These results collectively indicate a N-rich and P-limited regime of BHS and NYS, which is largely consistent with previous reports for these regions (Yang et al., 2018; Wang et al., 2019; Xin et al., 2019). Our measurements of surface Chl-*a* were generally low (<1 μg L^–1^) in winter and higher (>1 μg L^–1^) in summer, indicating a higher contribution of pico-/nanophytoplankton in the total phytoplankton biomass in the winter in the studied areas (Marañón et al., 2012). The characteristics of phytoplankton size classes in BHS and NYS have been demonstrated by *in situ* measurements of size-fractionated Chl-*a* and satellite-based observations (Sun D. et al., 2019; Sun X. et al., 2019). Nevertheless, our study contributes knowledge on the genetic diversity and taxonomic community composition, as well as, seasonal and spatial variability of P/NEs. Moreover, the trophic types (autotrophs, mixotrophs, and heterotrophs) of pico-/nanoeukaryotic components in the microbial loop of the studied areas are revealed.

### Operational Taxonomic Unit Richness of P/NEs Across Productivity and Dissolved Oxygen Gradients

The local and regional species richness and productivity relationship (SPR) is one of the central topics in community ecology. The curves describing SPR can be linear positive, hump-shaped (unimodal), linear negative, U-shaped, or of non-significant pattern. The unimodal relationship has been the most frequently recognized one in macroorganisms (Mittelbach et al., 2001; Scheiner and Willig, 2005; Whittaker, 2010) and marine phytoplankton (Vallina et al., 2014), while the U-shaped relationship has been found only rarely in microorganisms (Smith, 2007). Therefore, our observation of a U-shaped relationship between pico-/nanoeukaryotic richness and Chl-*a* is somewhat extraordinary. In this type of relationship, richness achieves a clear minimum at intermediate productivity levels. Nonetheless, the mechanisms underlying this pattern have yet to be understood. The U-shaped curve (Figure 2C) was mainly attributed to Syndiniales, ciliates and MASTs (Supplementary Figure 2), all of which comprise mostly heterotrophs. On the negative side of the curve, the OTU numbers of these three groups sharply decreased with Chl-*a*. This observation might be attributed to certain species getting increasingly dominant during the bloom, leading to a decrease in OTU richness temporarily. However, at the intermediate Chl-*a* levels, additional DOC released by phytoplankton could promote overgrowth of certain bacterial groups (for example, Gammaproteobacteria; Alonso-Sáez et al., 2009). This could lower diversity of bacterial communities, alter the overall digestibility of bacterioplankton (Gong et al., 2016), and in turn select for specific bacterivorous populations of ciliates and MAST grazers (Jürgens and Güde, 1994). On the positive side of the curve, whereby Chl-*a* is sustained at even higher levels, Dinophyceae are consistently present in high proportions in the communities (Supplementary Table 5), which might stimulate parasitic Syndiniales in pico-fractions (Chambouvet et al., 2008). Alternatively, the increased OTU richness of Syndiniales might be due to release of additional phylotypes into the pico-/nano-sized pool, when highly abundant infected micro-sized host cells burst. Thus, the top-down and bottom-up effects and species turnover of heterotrophs and mixotrophs seemed to determine the variation of richness of P/NEs along the Chl-*a* gradient in this study. This idea agrees well with Scheiner and Willig (2005), who proposed a theoretical framework to explain U-shaped patterns, i.e., the inflection point of the curve was a consequence of trade-off or shift in two dominating mechanisms that control the number of individuals.

Notably, DO had a stronger and negative relationship with the OTU richness of P/NEs than Chl-*a* in surface waters of the studied regions (Figures 2B,C), suggesting that oxygenation status reflects species richness of P/NEs more comprehensively than productivity. From a biological point of view, DO is usually correlated with Chl-*a* (i.e., a higher phytoplankton biomass and primary production tends to yield more photosynthetically produced O_2_ leading to higher level of DO in the water), and it is often difficult to disentangle the effects of these two factors. Nevertheless, the unimodal relationship between Chl-*a* and DO observed in our study (Figure 8E) indicates existence of an ecological feedback mechanism, whereby DO peaks at intermediate levels of Chl-*a*. Previous studies have shown that under highly eutrophic conditions (Chl-*a* > 3 μg L^–1^), both carbon fixation and O_2_ production were mainly contributed by microphytoplankton (Marañón et al., 2012), accompanied by release of a higher amount of DOC (Baines and Pace, 1991; Marañón et al., 2005). Under these conditions, productivity and oxygen consumption by heterotrophic prokaryotes was enhanced (Cole et al., 1988; Baines and Pace, 1991), thus lowering the DO levels in surrounding waters. Due to relatively larger sizes, protists have much higher apparent half-saturation constants for O_2_ uptake (Fenchel and Finlay, 2008). Thus, a high DO concentration might relieve the competitive pressure of heterotrophic eukaryotes against bacteria, leading to an increase of their proportions in biomass (Figure 8D) and contributing to low species richness. In addition to bacterioplankton, P/NEs could also contribute significantly to community respiration (Harrison, 1986; Hernandez-Ruiz et al., 2018). Therefore, the DO level reflects the balance between biological production and consumption of O_2_. These processes are involved in not only maintaining the standing stock of autotrophs and heterotrophs, but also their physiological activities, both of which are controlled by a range of environmental variables (e.g., light availability and temperature). Apart from these biological effects, physical processes (e.g., wind induced air-water interface exchange) and diel cycle (some of the samples herein were collected at night, when Chl-*a* level was relatively steady, but the DO was lower compared with that during daytime) are also among the controlling factors of DO dynamics (Hull et al., 2008). The observed stronger relationship of richness with DO rather than Chl-*a* in this study, along with the highly dynamic nature of DO in surface waters, suggests that DO is likely a more important environmental factor than productivity in reflecting the richness of P/NEs in coastal oceans. Nevertheless, more accurate measurements of Chl-*a* concentration (e.g., using high performance liquid chromatography), and direct determination of primary productivity using ^14^C method would be worth applying in the future to more accurately explore the richness-productivity relationship.

### Limiting Nutrients Drive the Functional Composition of Pico-/Nanoeukaryote Community

A number of studies have investigated the functional traits of protistan communities based on metabarcoding data of 18S rRNA genes (e.g., Charvet et al., 2014; de Vargas et al., 2015; Genitsaris et al., 2015; Ramond et al., 2019). Using a similar approach, our study indicated functional redundancy of P/NEs in the BHS and NYS. While the taxonomic community composition of P/NEs was significantly correlated with DO (Figures 4B,C) and differed between seasons (Table 1), the functional traits (trophic strategies) of the community (Photo%, Hetero%, and Mixo% in read proportion) did not follow any of these correlations and trends (Figures 8B,D). This observed decoupling contrasts with the study by Ramond et al. (2019), who found that the taxonomic and functional diversity of marine planktonic protists of pico-, nano- and micro-size classes in the Atlantic were tightly coupled. Compared with ours, their samples were collected from stations spanning a much larger geographic distance (over 3600 vs. 600 km) and latitude gradient; it is likely that the large scale of sampling resulted in higher variability of functional diversity, as has already been observed for taxonomic diversity (Martiny et al., 2006). Our sampling of surface waters [vs. surface, deep chlorophyll maximum (DCM) and mesopelagic zone] in the open ocean (vs. open water, estuaries, and upwelling) exhibited relatively low environmental heterogeneity (e.g., in terms of irradiance, organic carbon sources, and abundance and composition of bacterial food), which might account for the observed reduced functional variability of the pico-/nanoeukaryotic communities in this study.

Unexpectedly, the functional composition of pico-/nanoeukaryotic communities was significantly related to ${\text{PO}}_{4}^{3-}$, a limiting nutrient in the BHS and NYS basins (Figure 8C). Under conditions of P-deficit, mixotrophic eukaryotes can obtain nutrients by predating on bacteria (e.g., Nygaard and Tobiesen, 1993). Nevertheless, this switch to heterotrophy might come with a cost, in that mixotrophs might not be as competitive as strict heterotrophs in ingesting and digesting bacterial prey. When the level of ${\text{PO}}_{4}^{3-}$ increased beyond a threshold (e.g., 0.08 μM), the use of both inorganic and bacterial resources might have led to a distinct advantage of the mixotrophs over the heterotrophs. Our observation of the trade-off between mixotrophs and heterotrophs along the nutrient gradient is in line with the model predicted by Edwards (2019). Nevertheless, the percentage of phototrophs in the pico-/nanoeukaryotic community was not significantly correlated with ${\text{PO}}_{4}^{3-}$, which was likely due to their capability of using non-phosphorus membrane lipids for growth in the face of P-limitation (Van Mooy et al., 2009). While the concentration of ${\text{PO}}_{4}^{3-}$ was positively correlated with Chl-*a* in our samples, that of ${\text{SiO}}_{3}^{2-}$ had a negative correlation with Chl-*a*. This suggests that uptake of ${\text{SiO}}_{3}^{2-}$ by phytoplankton (especially large-celled diatoms) led to the low level of ${\text{SiO}}_{3}^{2-}$ observed in this study. Therefore, the lower percentage of autotrophs at lower ${\text{SiO}}_{3}^{2-}$ supply might be a consequence of immobilization and competition for Si between nano- and micro-sized diatoms.

## Data Availability Statement

The datasets presented in this study can be found in online repositories. The names of the repository/repositories and accession number(s) can be found in the article/Supplementary Material.

## Author Contributions

YW and GL analyzed the data. FS and JD performed the sample preparation, DNA extraction, and submission to sequencing. SZ, PZ, and XZ took part in cruises for sampling and obtaining metadata. EG and JG revised the manuscript. YW, GL, and JG wrote the manuscript. JG conceived the research and designed the experiment. All authors approved the final version of the manuscript and agreed to its submission for publication.

## Conflict of Interest

The authors declare that the research was conducted in the absence of any commercial or financial relationships that could be construed as a potential conflict of interest.
