# Supplementary material for: Taxonomic Diversity of Pico-/Nanoeukaryotes Is Related to Dissolved Oxygen and Productivity, but Functional Composition Is Shaped by Limiting Nutrients in Eutrophic Coastal Oceans
Source: Front Microbiol. 2020 Dec 3;11:601037. doi: 10.3389/fmicb.2020.601037 (PMC7744618; doi:10.3389/fmicb.2020.601037)
Supplement: Supplementary file 1 [file Data_Sheet_1.PDF]

Supplementary Material

Supplementary Figures

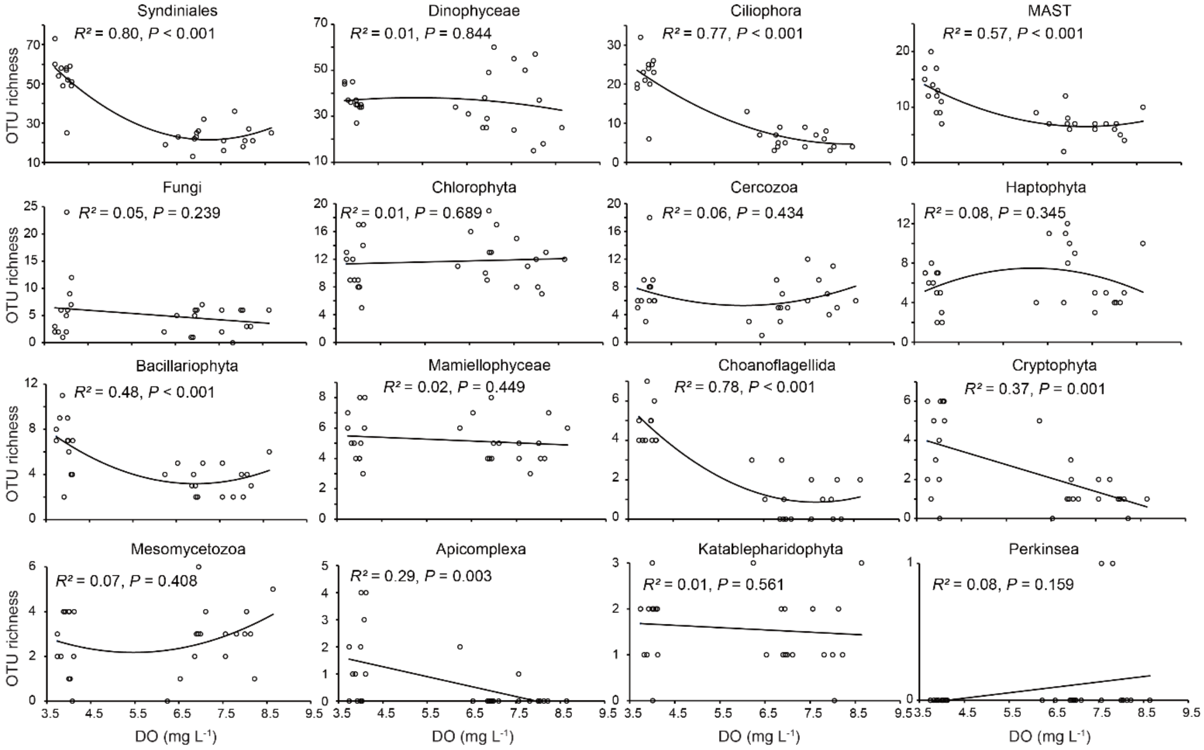

Supplementary Figure 1. Scatter plots showing the relationships between OTU numbers of major taxa and the concentration of dissolved oxygen. Note that those of the OTU-rich taxa Syndiniales, Ciliophora, and MAST were significantly U-shaped.

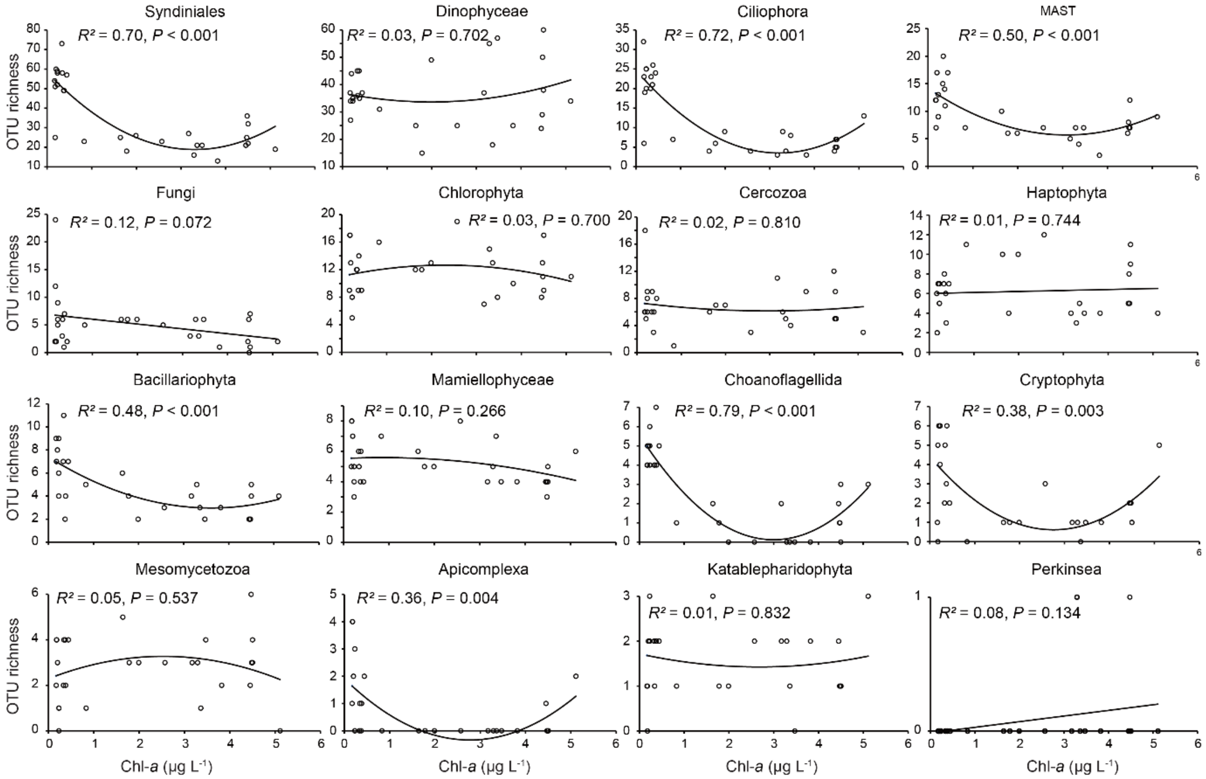

Supplementary Figure 2. Scatter plots showing the relationships between OTU numbers of major taxa and the concentration of chlorophyll a. Note that those of the OTU-rich taxa Syndiniales, Ciliophora, and MAST were significantly U-shaped.
