# Supplementary material for: Taxonomic Diversity of Pico-/Nanoeukaryotes Is Related to Dissolved Oxygen and Productivity, but Functional Composition Is Shaped by Limiting Nutrients in Eutrophic Coastal Oceans
Source: Front Microbiol. 2020 Dec 3;11:601037. doi: 10.3389/fmicb.2020.601037 (PMC7744618; doi:10.3389/fmicb.2020.601037)
Supplement: Supplementary file 2 [file Data_Sheet_2.pdf]

Table S1. Summary of sampling and environmental variables of water samples collected from the BHS and NYS in 2011.

**Sample ID**	**Latitude (N)**	**Longitude (E)**	**Date (mm/dd)**	**Temp (℃)**	**DO (mg L^{-1})**	**Sal (PSU)**	**Chl-*a* (μg L^{-1})**	**NO_3^- (μM)**	**NO_2^- (μM)**	**NH_4^+ (μM)**	**DIN (μM)**	**DON (μM)**	**PO_4^{3-} (μM)**	**SiO_3^{2-} (μM)**	**N/P**	**N/Si**	**P/Si**
BH40S	38°20.387′	120°27.087′	6/24	14.36	8.13	30.97	3.17	0.89	0.18	5.09	6.16	11.55	0.05	1.43	123.2	4.3	0.03
BH41S	38°20.079′	120°12.018′	6/24	15.70	7.81	31.03	4.48	0.93	0.17	7.54	8.64	5.15	0.07	0.82	123.4	10.5	0.09
BH42S	38°19.886′	119°46.958′	6/25	19.01	6.86	30.64	3.82	2.46	0.24	11.40	14.10	11.26	0.04	0.57	352.5	24.7	0.07
BH45S	38°19.102′	118°59.834′	6/25	19.43	7.56	31.06	4.45	2.67	0.28	3.64	6.60	9.69	0.08	2.11	82.5	3.1	0.04
BH47S	38°40.008′	118°58.401′	6/25	17.67	8.00	31.25	1.78	1.14	0.28	9.80	11.22	7.85	0.07	1.21	160.3	9.3	0.06
BH60S	39°19.164′	120°14.321′	6/26	17.92	6.90	31.21	4.50	0.89	0.26	7.08	8.22	11.14	0.09	2.64	91.3	3.1	0.03
BH68S	37°44.319′	119°45.341′	6/28	21.47	6.24	30.85	5.11	2.05	0.30	0.41	2.76	8.38	0.14	1.54	19.7	1.8	0.09
NY10S	36°59.060′	123°59.552′	6/21	21.03	6.53	31.95	0.83	0.89	0.20	8.86	9.95	9.62	0.04	1.00	248.8	10.0	0.04
NY12S	37°53.679′	123°03.585′	6/22	18.93	6.99	31.63	1.99	0.75	0.21	8.23	9.19	7.81	0.07	0.57	131.3	16.1	0.12
NY15S	38°44.419′	123°42.903′	6/22	19.42	6.94	31.79	2.57	1.40	0.37	13.94	15.70	10.01	0.09	0.89	174.4	17.6	0.10
NY22S	38°44.874′	122°29.992′	6/22	15.50	8.22	31.01	3.36	0.81	0.21	9.86	10.87	4.99	0.09	0.86	120.8	12.6	0.10
NY24S	38°09.409′	122°29.021′	6/23	18.66	7.11	31.22	4.50	1.14	0.40	4.39	5.93	21.86	0.13	1.21	45.6	4.9	0.11
NY28S	37°42.011′	121°59.491′	6/23	13.15	8.64	31.48	1.64	0.71	0.23	10.48	11.42	6.22	0.09	0.25	126.9	45.7	0.36
NY30S	38°11.923′	121°59.871′	6/24	18.97	6.95	31.18	4.47	0.80	0.26	10.90	11.96	8.75	0.09	0.71	132.9	16.9	0.13
NY36S	38°16.043′	121°16.042′	6/24	17.29	7.56	30.58	3.29	0.90	0.26	5.82	6.98	13.23	0.10	1.54	69.8	4.5	0.06
NY38S	37°54.656′	121°09.491′	6/24	15.25	8.04	31.18	3.47	0.76	0.20	13.53	14.49	4.80	0.07	0.29	207.0	50.0	0.24
BH40W	38°20.316′	120°27.152′	11/25	13.05	4.00	31.10	0.23	2.56	0.60	14.93	18.09	17.62	0.03	3.07	603.0	5.9	0.01
BH41W	38°20.133′	120°11.523′	11/25	12.71	4.08	31.20	0.23	1.18	0.72	6.32	8.21	10.08	0.07	0.82	117.3	10.0	0.09
BH42W	38°19.733′	119°47.043′	11/26	12.79	4.03	30.87	0.23	3.60	0.25	2.80	6.65	15.42	0.05	6.96	133.0	1.0	0.01
BH45W	38°19.090′	118°59.975′	11/26	12.26	4.01	30.07	0.18	3.57	1.03	18.20	22.79	16.71	0.08	2.61	284.9	8.7	0.03
NY12W	37°53.693′	123°03.726′	11/22	13.67	3.92	30.88	0.37	1.37	0.54	57.99	59.90	18.24	0.06	3.04	998.3	19.7	0.02
NY15W	38°44.509′	123°43.133′	11/22	12.21	3.88	31.65	0.34	2.13	0.39	127.71	130.2	2.98	0.06	2.89	2170.5	45.1	0.02
NY22W	38°44.862′	122°29.950′	11/24	12.09	3.74	31.03	0.33	2.38	0.42	13.22	16.02	10.12	0.06	4.21	267.0	3.8	0.01

NY24W	38°09.764′	122°29.276′	11/24	12.39	4.00	31.03	0.44	1.74	0.35	5.12	7.20	6.87	0.06	3.86	120.0	1.9	0.02
NY28W	37°42.049′	121°59.563′	11/24	12.13	4.11	30.97	0.38	2.84	0.44	3.41	6.69	12.24	0.07	6.29	95.6	1.1	0.01
NY30W	38°11.945′	121°39.592′	11/24	13.42	3.74	30.89	0.20	2.81	0.55	15.67	19.04	16.03	0.07	4.14	272.0	4.6	0.02
NY36W	38°15.958′	121°16.020′	11/25	12.99	3.82	30.97	0.17	3.16	0.62	2.66	6.44	25.35	0.10	8.93	64.4	0.7	0.01
NY38W	37°54.632′	121°09.594′	11/25	12.50	4.12	30.93	0.18	2.48	0.34	11.47	14.28	11.08	0.09	4.82	158.7	3.0	0.02

Table S2. Functional assignments for taxonomic groups of eukaryotes

Taxonomy	Functional trait
Amoebozoa	Heterotrophic
Apusozoa	Heterotrophic
Alveolata; Apicomplexa	Heterotrophic
Alveolata; Ciliophora	Heterotrophic
Alveolata; Perkinsea	Heterotrophic
Hacrobia; Centroheliozoa	Heterotrophic
Hacrobia; Katablepharidophyta	Heterotrophic
Hacrobia; Picobiliphyta	Heterotrophic
Hacrobia; Telonemia	Heterotrophic
Opisthokonta; Choanoflagellida	Heterotrophic
Opisthokonta; Fungi	Heterotrophic
Opisthokonta;Mesomycetozoa	Heterotrophic
Rhizaria;Cercozoa	Heterotrophic
Alveolata; Dinophyta; Syndiniales	Heterotrophic
Stramenopiles; Stramenopiles_X; Bicoecea	Heterotrophic
Stramenopiles; Stramenopiles_X; Labyrinthulea	Heterotrophic
Stramenopiles; Stramenopiles_X; MAST	Heterotrophic
Stramenopiles; Stramenopiles_X; Oomycota	Heterotrophic
Stramenopiles; Stramenopiles_X; Pirsonia	Heterotrophic
Archaeplastida	Phototrophic
Stramenopiles; Stramenopiles_X; MOCH	Phototrophic
Stramenopiles; Ochrophyta	Phototrophic
Hacrobia; Cryptophyta	Mixotrophic
Hacrobia; Haptophyta	Mixotrophic
Alveolata; Dinophyta; Dinophyceae	Mixotrophic

Table S3. Summary of pyrotag processing and OTU richness of pico-nanoplanktonic microeukaryotes in the surface waters of BHS and NYS.

Basin/season	Sample ID	Barcode sequence	Raw seqs	Quality seqs	OTU richness (1590)
BHS/ Summer	BH40S	ATACGACGTA	3832	2227	114
	BH41S	CGTCTAGTAC	5701	3967	140
	BH42S	TCTACGTAGC	3999	2623	86
	BH45S	TGTACTACTC	7691	4558	127
	BH47S	ACGACTACAG	9493	6632	100
	BH60S	CGTAGACTAG	7741	5181	122
	BH68S	TACGAGTATG	4420	2482	132
NYS/ Summer	NY10S	AGCACTGTAG	4266	2145	125
	NY12S	ATCAGACACG	4593	2086	161
	NY15S	ATATCGCGAG	6000	3151	130
	NY22S	CGTGTCTCTA	4647	2578	90
	NY24S	CTCGCGTGTC	6982	4158	174
	NY28S	TAGTATCAGC	7232	4863	120
	NY30S	TACTGAGCTA	3483	1596	126
	NY36S	CATAGTAGTG	3537	1852	148
	NY38S	CGAGAGATAC	4131	2590	130
BHS/ Winter	BH40W	TACACACACT	3568	1663	192
	BH41W	TACAGATCGT	4663	2428	209
	BH42W	TACGCTGTCT	9501	6565	178
	BH45W	TAGTGTAGAT	12330	4986	172
NYS/ Winter	NY12W	ACGCGAGTAT	3736	2492	178
	NY15W	ACTACTATGT	4604	2420	233
	NY22W	ACTGTACAGT	4276	2075	224
	NY24W	AGACTATACT	4825	2470	190
	NY28W	AGCGTCGTCT	6481	3992	192
	NY30W	CACGCTACGT	3476	1984	214
	NY36W	CAGTAGACGT	5423	3154	198
	NY38W	CGACGTGACT	15250	7724	198

Table S4. Shifts in relative proportion (%, mean ±SE) of pico-nanoplanktonic eukaryotic lineages in the sequencing data for the surface waters between seasons (summer and winter), basins (BHS and NYS) in Bohai Sea and North Yellow Sea.

Taxon	**Season**		**Basin**	
	**Summer (n = 16)**	**Winter (n = 12)**	**BHS (n = 11)**	**NYS (n = 17)**
ALVEOLATA	59.8 ±3.68	62.7 ±2.63	59.6 ±3.85	62.0 ±3.06
Dinophyta	58.4 ±3.87	56.0 ±2.69	56.1 ±4.14	58.2 ±3.13
Dinophyceae	34.2 ±3.63	32.2 ±3.94	28.5 ±1.99	36.5 ±4.02
Dinophysiales	0.0 ±0.01	0.0 ±0.00	0.0 ±0.00	0.0 ±0.01
Suessiales	7.2 ±0.92[a]	1.9 ±0.50[b]	5.2 ±1.27	4.8 ±0.96
Syndiniales	24.1 ±3.93	23.8 ±2.17	27.6 ±3.88	21.7 ±3.00
Dino-Group-I	20.5 ±3.94	10.8 ±2.19	19.6 ±4.12	14.3 ±3.30
Dino-Group-II	3.4 ±0.44[b]	12.1 ±1.82[a]	7.4 ±2.24	7.0 ±1.29
Dino-Group-III	0.2 ±0.05[b]	0.8 ±0.23[a]	0.6 ±0.28	0.3 ±0.08
Dino-Group-IV	0.0 ±0.00[b]	0.1 ±0.02[a]	0.0 ±0.00[b]	0.0 ±0.02[a]
Ciliophora	1.4 ±0.55[b]	6.5 ±1.02[a]	3.4 ±1.17	3.7 ±0.94
Spirotrichea	1.2 ±0.54[b]	5.0 ±0.83[a]	2.7 ±0.96	2.9 ±0.77
Choreotrichia	0.5 ±0.36[b]	1.5 ±0.25[a]	1.2 ±0.57	0.8 ±0.20
Oligotrichia	0.7 ±0.19[b]	3.5 ±0.65[a]	1.5 ±0.46	2.1 ±0.58
Colpodea	0.1 ±0.03[b]	0.6 ±0.16[a]	0.4 ±0.16	0.3 ±0.10
Litostomatea	0.1 ±0.02[b]	0.7 ±0.16[a]	0.3 ±0.15	0.4 ±0.12
Cyclotrichia	0.1 ±0.02[b]	0.6 ±0.14[a]	0.2 ±0.11	0.4 ±0.11
Haptoria	0.0 ±0[b]	0.1 ±0.04[a]	0.0 ±0.04	0.0 ±0.01
Oligohymenophorea	0.0 ±0.00[b]	0.1 ±0.02[a]	0.0 ±0.02	0.1 ±0.02
Scuticociliatia	0.0 ±0.0	0.0 ±0.02	0.0 ±0.0	0.0 ±0.02
Phyllopharyngea	0.0 ±0.00	0.0 ±0.01	0.0 ±0.00	0.0 ±0.00
Cyrtophoria	0.0 ±0.0	0.0 ±0.01	0.0 ±0.0	0 ±0.01
Suctoria	0.0 ±0.0	0.0 ±0.01	0.0 ±0.0	0.0 ±0.0
Apicomplexa	0.0 ±0.02 [b]	0.3 ±0.09 [a]	0.1 ±0.07	0.1 ±0.06
HACROBIA	11.6 ±3.11	10.1 ±1.14	10.5 ±3.24	11.2 ±2.23
Haptophyta	6.3 ±1.80	3.0 ±0.49	3.6 ±0.90	5.7 ±1.68
Prymnesiophyceae/ Isochrysidales	3.0 ±0.86	1.4 ±0.31	2.1 ±0.64	2.4 ±0.78
Cryptophyta	2.6 ±1.92[b]	5.5 ±0.81[a]	5.0 ±2.74	3.1 ±0.80
Katablepharidophyta	1.2 ±0.30[a]	0.5 ±0.16[b]	1.0 ±0.21	0.8 ±0.30
Picobiliphyta	0.3 ±0.15[b]	0.6 ±0.10[a]	0.5 ±0.23	0.4 ±0.09
Telonemia	0.9 ±0.30	0.6 ±0.05	0.4 ±0.11	0.9 ±0.27
OPISTHOKONTA	10.2 ±2.36[a]	3.2 ±0.65[b]	7.6 ±2.99	6.9 ±1.65
Mesomycetozoa	9.6 ±2.36[a]	0.9 ±0.25[b]	5.8 ±3.13	5.9 ±1.69
Ichthyosporea	9.6 ±2.36[a]	0.9 ±0.25[b]	5.8 ±3.13	5.9 ±1.69
Fungi	0.4 ±0.10	1.4 ±0.6	1.2 ±0.67	0.6 ±0.11
Ascomycota	0.2 ±0.05	0.8 ±0.44	0.8 ±0.48	0.2 ±0.05
Basidiomycota	0.1 ±0.03	0.2 ±0.09	0.2 ±0.09	0.1 ±0.04
Chytridiomycota	0.1 ±0.03[b]	0.3 ±0.07[a]	0.2 ±0.08	0.2 ±0.04
Cryptomycota	0.0 ±0.03	0.0 ±0.02	0.0 ±0.02	0.0 ±0.03

Choanoflagellida	0.1 ±0.02[b]	0.7 ±0.17[a]	0.5 ±0.22	0.2 ±0.06
Choanoflagellatea	0.1 ±0.02[b]	0.4 ±0.08[a]	0.3 ±0.11	0.2 ±0.04
Acanthoecida	0.0 ±0.01[b]	0.3 ±0.07[a]	0.2 ±0.09	0.1 ±0.03
STRAMENOPILES	9.7 ±1.94	10.7 ±1.66	12.4 ±2.94	8.7 ±0.92
Ochrophyta	5.3 ±1.90	5.8 ±1.21	7.6 ±2.73	4.2 ±0.78
Pelagophyceae	3.3 ±1.84	2.0 ±0.5	4.3 ±2.58	1.7 ±0.55
Bacillariophyta	1.4 ±0.30	3.3 ±1.04	2.7 ±1.06	1.9 ±0.47
Chrysophyceae-Synurophyceae	0.3 ±0.10	0.2 ±0.03	0.1 ±0.04	0.3 ±0.09
Dictyochophyceae	0.1 ±0.04	0.1 ±0.03	0.1 ±0.04	0.1 ±0.04
Bolidophyceae & relatives	0.0 ±0.01[b]	0.2 ±0.05[a]	0.1 ±0.05	0.1 ±0.03
Raphidophyceae	0.1 ±0.03	0.1 ±0.03	0.1 ±0.04	0.1 ±0.02
Stramenopiles_X	4.4 ±0.54	4.9 ±0.66	4.8 ±0.69	4.5 ±0.52
Bicoecea	0.2 ±0.10	0.1 ±0.03	0.2 ±0.14	0.1 ±0.03
Labyrinthulea	0.4 ±0.09	0.4 ±0.16	0.4 ±0.18	0.4 ±0.09
Labyrinthulales	0.4 ±0.08	0.4 ±0.15	0.4 ±0.17	0.4 ±0.08
Thraustochytriales	0 ±0.02	0 ±0.01	0 ±0.01	0 ±0.02
MAST	3.4 ±0.50	3.3 ±0.41	2.7 ±0.31	3.7 ±0.49
MAST-1	1.4 ±0.39	0.8 ±0.23	0.7 ±0.23	1.3 ±0.37
MAST-3	0.6 ±0.14	0.8 ±0.15	0.7 ±0.17	0.7 ±0.13
MAST-4	0.0 ±0.03[b]	0.5 ±0.10[a]	0.2 ±0.08	0.3 ±0.09
MAST-7	0.0 ±0.01[b]	0.3 ±0.09[a]	0.0 ±0.01	0.2 ±0.08
MAST-8	0.1 ±0.04	0.1 ±0.03	0 ±0.02[b]	0.1 ±0.03[a]
MAST-9	0.5 ±0.10[a]	0.2 ±0.13[b]	0.5 ±0.14	0.3 ±0.10
MAST-10	0.0 ±0.00[b]	0.1 ±0.02[a]	0.0 ±0.01	0.0 ±0.02
MOCH	0.2 ±0.11	0.0 ±0.01	0.3 ±0.16	0.0 ±0.01
Oomycota	0.1 ±0.04[b]	0.8 ±0.32[a]	0.8 ±0.36	0.1 ±0.04
Pirsonia	0.0 ±0.02	0.2 ±0.14	0.2 ±0.15	0.0 ±0.02
ARCHAEPLASTIDA	6.1 ±1.20[b]	11.1 ±1.36[a]	5.7 ±1.19[b]	9.9 ±1.34[a]
Chlorophyta	6.1 ±1.20[b]	11.1 ±1.36a	5.7 ±1.19[b]	9.9 ±1.34[a]
Mamiellophyceae	1.7 ±0.21[b]	8.0 ±1.13[a]	2.9 ±0.76[b]	5.3 ±1.12 [a]
Dolichomastigales	0.2 ±0.03[a]	0.0 ±0.01[b]	0.1 ±0.04	0.1 ±0.02
Mamiellales	1.5 ±0.21[b]	7.9 ±1.13[a]	2.8 ±0.78	5.2 ±1.14
Prasino-Clade-I	0.0 ±0.00	0.0 ±0.01	0.0 ±0.00	0.0 ±0.00
Prasino-Clade-V	0.3 ±0.08	0.2 ±0.05	0.1 ±0.03[b]	0.3 ±0.07[a]
Prasinococcales	0.0 ±0.01	0.0 ±0.01	0.0 ±0.01	0.0 ±0.01
Nephroselmidophyceae	0.1 ±0.03	0.1 ±0.03	0.1 ±0.04	0.1 ±0.03
Pyramimonadales	1.8 ±0.3	1.4 ±0.53	1.7 ±0.49	1.6 ±0.34
RHIZARIA	2.4 ±0.67	1.9 ±0.55	3.8 ±0.92[a]	1.2 ±0.21[b]
Cercozoa	2.4 ±0.67	1.9 ±0.55	3.8 ±0.92[a]	1.2 ±0.21[b]
Filosa-Chlorarachnea	0.0 ±0.00	0.1 ±0.03	0.0 ±0.03	0.0 ±0.02
Filosa-Thecofilosea	2.4 ±0.67	1.8 ±0.55	3.7 ±0.92[a]	1.2 ±0.21[b]
AMOEBOZOA	0.1 ±0.02	0.1 ±0.04	0.1 ±0.05[a]	0.0 ±0.01[b]
APUSOZOA	0.1 ±0.06	0.1 ±0.03	0.2 ±0.09[a]	0.0 ±0.02[b]
Apusomonadidae	0.0 ±0.01	0.1 ±0.03	0.1 ±0.02	0.0 ±0.02

Significant differences ($P \leq 0.05$) were indicated with different superscript letters.

Table S5. Shifts in relative proportion (%, mean ±SE) of pico-nanoplanktonic eukaryotic lineages across levels of dissolved oxygen and chlorophyll *a*.

**Taxon**	**Dissolved oxygen**			**Chlorophyll *a***		
	**3 - 5 mg L^{-1} (n=12)**	**5 - 7 mg L^{-1} (n=7)**	**7 - 9 mg L^{-1} (n=9)**	**0 - 1 mg L^{-1} (n=13)**	**1 - 4 mg L^{-1} (n=9)**	**4 - 6 mg L^{-1} (n=6)**
ALVEOLATA	62.7 ±2.63	53.2 ±6.97	64.9 ±3.04	62.9 ±2.43	59.7 ±5.73	59.0 ±5.32
Dinophyta	56.0 ±2.69	50.8 ±7.3	64.3 ±2.97	56.6 ±2.57	59 ±5.73	56.4 ±6.22
Dinophyceae	32.2 ±3.94	28.5 ±4.23	38.7 ±5.27	30.8 ±3.88	34.3 ±5.63	37.5 ±3.75
Dinophysiales	0.0 ±0.00	0.0 ±0.01	0.0 ±0.02	0.0 ±0.00	0.0 ±0.01	0.0 ±0.03
Suessiales	1.9 ±0.50[b]	5.9 ±1.02[a]	8.2 ±1.38[a]	2.2 ±0.51[b]	8.3 ±1.45[a]	6.0 ±0.90[a]
Syndiniales	23.8 ±2.17	22.3 ±7.13	25.5 ±4.62	25.9 ±2.87	24.6 ±5.17	19.0 ±5.56
Dino-Group-I	10.8 ±2.19	18.6 ±7.00	22.0 ±4.75	13.7 ±3.51	20.5 ±5.08	16.0 ±5.69
Dino-Group-II	12.1 ±1.82[a]	3.4 ±0.69[b]	3.5 ±0.61[b]	11.3 ±1.87[a]	4.1 ±0.58[b]	2.8 ±0.64[b]
Dino-Group-III	0.8 ±0.23[a]	0.3 ±0.11[b]	0.1 ±0.02[b]	0.8 ±0.21[a]	0.1 ±0.02[b]	0.2 ±0.04[b]
Dino-Group-IV	0.1 ±0.02[a]	0.0 ±0.00[b]	0.0 ±0.00[b]	0.1 ±0.02[a]	0.0 ±0.00[b]	0.0 ±0.00[b]
Ciliophora	6.5 ±1.02[a]	2.3 ±1.20[b]	0.6 ±0.11[b]	6.1 ±1.04[a]	0.7 ±0.15[b]	2.4 ±1.42[b]
Spirotrichea	5.0 ±0.83[a]	2.1 ±1.20[b]	0.5 ±0.09[b]	4.7 ±0.84[a]	0.6 ±0.13[b]	2.1 ±1.42[ab]
Choreotrichia	1.5 ±0.25[a]	1.0 ±0.82[ab]	0.1 ±0.03[b]	1.4 ±0.25	0.1 ±0.04	1.1 ±0.96
Choreotrichia-1	0.5 ±0.16[a]	0.0 ±0.01[b]	0.0 ±0.01[b]	0.5 ±0.15[a]	0.0 ±0.01[b]	0.0 ±0.00[b]
Lynnellidae	0.0 ±0.03	0.1 ±0.07	0.0 ±0.01	0.0 ±0.03	0.0 ±0.01	0.1 ±0.08
Strobilidiidae	0.4 ±0.12	0.5 ±0.44	0.0 ±0.01	0.4 ±0.11	0.0 ±0.02	0.6 ±0.51
Strombidinopsidae	0.0 ±0.02	0.1 ±0.14	0.0 ±0.00	0.0 ±0.02	0.0 ±0.00	0.2 ±0.17
Tintinnidae	0.1 ±0.01	0.2 ±0.18	0.0 ±0.03	0.1 ±0.01	0.0 ±0.03	0.2 ±0.21
Tintinnidiidae	0.0 ±0.01	0.0 ±0.00	0.0 ±0.00	0.0 ±0.01	0.0 ±0.00	0.0 ±0.00
Oligotrichia	3.5 ±0.65[a]	1.1 ±0.39[b]	0.4 ±0.08[b]	3.2 ±0.64[a]	0.5 ±0.12[b]	1.0 ±0.48[b]
Strombidiidae	2.7 ±0.53[a]	0.8 ±0.31[b]	0.3 ±0.08[b]	2.5 ±0.52[a]	0.4 ±0.10[b]	0.8 ±0.37[b]
Litostomatea	0.7 ±0.16[a]	0.1 ±0.03[b]	0.1 ±0.03[b]	0.7 ±0.16[a]	0.1 ±0.03[b]	0.1 ±0.03[b]

Colpodea	0.6 ±0.16[a]	0.1 ±0.07[b]	0.0 ±0.01[b]	0.6 ±0.16[a]	0.0 ±0.01[b]	0.1 ±0.08[b]
Oligohymenophorea	0.1 ±0.02[a]	0.0 ±0.01[b]	0.0 ±0.00[b]	0.1 ±0.02[a]	0.0 ±0.01[b]	0.0 ±0.00[b]
Phyllopharyngea	0.0 ±0.01	0.0 ±0.00	0.0 ±0.00	0.0 ±0.01	0.0 ±0.00	0.0 ±0.00
Apicomplexa	0.3 ±0.09	0.1 ±0.05	0.0 ±0.02	0.2 ±0.09	0.0 ±0.00	0.1 ±0.06
HACROBIA	10.1 ±1.14[b]	21 ±5.23[a]	4.3 ±0.98[b]	11.0 ±1.36	9.0 ±4.24	13.8 ±5.27
Cryptophyta	5.5 ±0.81	5.6 ±4.28	0.3 ±0.08	5.1 ±0.85	0.6 ±0.39	6.0 ±5.02
Cryptophyceae	5.5 ±0.81	5.6 ±4.28	0.3 ±0.08	5.1 ±0.85	0.6 ±0.39	6.0 ±5.02
Haptophyta	3.0 ±0.49[b]	10.9 ±3.32[a]	2.8 ±0.85[b]	4.0 ±1.17	5.7 ±2.87	5.4 ±1.41
Pavlovophyceae	0.0 ±0.01	1.2 ±1.05	0.7 ±0.57	0.0 ±0.01	1.5 ±0.94	0.1 ±0.07
Pavlovales	0.0 ±0.01	1.2 ±1.05	0.7 ±0.57	0.0 ±0.01	1.5 ±0.94	0.1 ±0.07
Prymnesiophyceae	2.9 ±0.49[b]	9.7 ±2.51[a]	2.1 ±0.76[b]	4.0 ±1.17	4.2 ±2.14	5.3 ±1.39
Isochrysidales	1.4 ±0.31[b]	5.6 ±1.37[a]	1.0 ±0.49[b]	1.9 ±0.58	2.4 ±1.27	3.0 ±1.10
Phaeocystales	0.2 ±0.07	0.4 ±0.12	0.1 ±0.06	0.3 ±0.07	0.1 ±0.09	0.3 ±0.12
Prymnesiales	1.3 ±0.24[b]	3.5 ±1.07[a]	1.0 ±0.39[b]	1.9 ±0.58	1.5 ±0.70	2.0 ±0.48
Katablepharidophyta	0.5 ±0.16[b]	1.9 ±0.55[a]	0.6 ±0.20[b]	0.5 ±0.15	1.2 ±0.52	1.3 ±0.27
Picobiliphyta	0.6 ±0.10[a]	0.5 ±0.34[a]	0.1 ±0.02[b]	0.6 ±0.09	0.1 ±0.03	0.5 ±0.41
Telonemia	0.6 ±0.05[b]	1.4 ±0.63[a]	0.4 ±0.13[b]	0.6 ±0.09	1.1 ±0.52	0.4 ±0.11
OPISTHOKONTA	3.2 ±0.65[b]	6.1 ±2.79[b]	13.3 ±3.34[a]	3.1 ±0.62[b]	12.9 ±3.46[a]	7.6 ±3.12[ab]
Mesomycetozoa	0.9 ±0.25[b]	5.6 ±2.73[b]	12.8 ±3.37[a]	0.9 ±0.23[a]	12.2 ±3.46[b]	7.2 ±3.15[b]
Ichthyosporea/Ichthyosphonida	0.9 ±0.25[b]	5.6 ±2.73[b]	12.8 ±3.37[a]	0.9 ±0.23[a]	12.2 ±3.46[b]	7.2 ±3.15[b]
Fungi	1.4 ±0.60	0.4 ±0.14	0.5 ±0.13	1.3 ±0.56	0.6 ±0.12	0.3 ±0.17
Ascomycota	0.8 ±0.44	0.2 ±0.07	0.2 ±0.06	0.7 ±0.41	0.3 ±0.07	0.1 ±0.06
Basidiomycota	0.2 ±0.09	0.1 ±0.04	0.1 ±0.05	0.2 ±0.08	0.2 ±0.05	0.1 ±0.03
Chytridiomycota	0.3 ±0.07[a]	0.1 ±0.07[ab]	0.1 ±0.03[b]	0.3 ±0.07[a]	0.1 ±0.06[ab]	0.0 ±0.01[b]
Cryptomycota	0.0 ±0.00	0.0 ±0.03	0.1 ±0.06	0.0 ±0.00	0.0 ±0.03	0.1 ±0.08

Mucoromycota	0.0 ±0.01	0.0 ±0.00	0.0 ±0.00	0.0 ±0.01	0.0 ±0.00	0.0 ±0.00
Choanoflagellida	0.7 ±0.17[a]	0.1 ±0.04[b]	0.1 ±0.02[b]	0.6 ±0.17[a]	0.0 ±0.02[b]	0.1 ±0.04[b]
Choanoflagellatea	0.4 ±0.08[a]	0.1 ±0.04[b]	0.0 ±0.01[b]	0.4 ±0.08[a]	0.0 ±0.01[b]	0.1 ±0.04[b]
Acanthoecida	0.3 ±0.07[a]	0.0 ±0.02[b]	0.0 ±0.01[b]	0.3 ±0.06[a]	0.0 ±0.01[b]	0.1 ±0.01[b]
STRAMENOPILES	10.7 ±1.66	12.7 ±4.13	7.4 ±1.03	10.4 ±1.55	8.0 ±1.22	12.7 ±4.86
Ochrophyta	5.8 ±1.21	7.6 ±4.22	3.5 ±0.87	5.6 ±1.12	3.9 ±1.07	7.7 ±4.93
Pelagophyceae	2.0 ±0.50	5.5 ±4.09	1.5 ±0.81	1.8 ±0.47	2.0 ±1.03	5.7 ±4.75
Bacillariophyta	3.3 ±1.04	1.2 ±0.29	1.5 ±0.50	3.1 ±0.96	1.4 ±0.49	1.3 ±0.37
Chrysophyceae-Synurophyceae	0.2 ±0.03[b]	0.6 ±0.18[a]	0.1 ±0.03[b]	0.2 ±0.09	0.2 ±0.12	0.2 ±0.09
Dictyochophyceae	0.1 ±0.03[b]	0.3 ±0.08[a]	0.0 ±0.01[b]	0.1 ±0.02	0.1 ±0.06	0.1 ±0.07
Raphidophyceae	0.1 ±0.03	0.0 ±0.01	0.1 ±0.06	0.1 ±0.03	0.1 ±0.06	0.0 ±0.01
Eustigmatophyceae	0.0 ±0.01	0.0 ±0.01	0.2 ±0.20	0.0 ±0.01	0.0 ±0.01	0.3 ±0.29
Bolidophyceae-and-relatives	0.2 ±0.05[a]	0.0 ±0.02[b]	0.0 ±0.00[b]	0.2 ±0.05[a]	0.0 ±0.00[b]	0.0 ±0.01[b]
Stramenopiles_X	4.9 ±0.66	5.1 ±0.71	3.9 ±0.77	4.8 ±0.62	4.1 ±0.93	5.0 ±0.31
MAST	3.3 ±0.41	3.9 ±0.75	3.0 ±0.68	3.2 ±0.38	3.3 ±0.9	3.6 ±0.18
MAST-1	0.8 ±0.23	2.0 ±0.80	0.8 ±0.24	0.7 ±0.21	1.4 ±0.64	1.5 ±0.42
MAST-9	0.2 ±0.13	0.4 ±0.09	0.6 ±0.15	0.2 ±0.12	0.5 ±0.11	0.6 ±0.20
MAST-4	0.5 ±0.10[a]	0.1 ±0.08[b]	0 ±0.01[b]	0.4 ±0.10[a]	0.0 ±0.01[b]	0.1 ±0.09[b]
MAST-7	0.3 ±0.09[a]	0.0 ±0.02[b]	0.0 ±0.00[b]	0.3 ±0.09[a]	0.0 ±0.00[b]	0.0 ±0.01[b]
MAST-10	0.1 ±0.02[a]	0.0 ±0.01[b]	0.0 ±0.00[b]	0.1 ±0.02[a]	0.0 ±0.00[b]	0.0 ±0.01[b]
Labyrinthulea	0.4 ±0.16	0.3 ±0.10	0.4 ±0.15	0.4 ±0.15	0.4 ±0.13	0.4 ±0.16
Bicoecea	0.1 ±0.03[b]	0.4 ±0.2[a]	0.1 ±0.02[b]	0.1 ±0.03[b]	0.1 ±0.03[b]	0.4 ±0.23[a]
MOCH	0.0 ±0.01	0.3 ±0.16	0.2 ±0.16	0.0 ±0.01[b]	0.1 ±0.08[b]	0.5 ±0.26[a]
Oomycota	0.8 ±0.32	0.1 ±0.10	0.1 ±0.03	0.7 ±0.30	0.1 ±0.07	0.0 ±0.01
Pirsonia	0.2 ±0.14	0 ±0.02	0.1 ±0.03	0.2 ±0.13	0.0 ±0.02	0.1 ±0.04
ARCHAEPLASTIDA	11.1 ±1.36[a]	5.2 ±1.07[b]	6.8 ±2.00[b]	10.7 ±1.34[a]	7.3 ±2.06[ab]	4.4 ±0.40[b]

Chlorophyta	11.1 ± 1.36[a]	5.2 ± 1.07[b]	6.8 ± 2.00[b]	10.7 ± 1.34[a]	7.3 ± 2.06[ab]	4.4 ± 0.40[b]
Mamiellophyceae	8.0 ± 1.13[a]	2.0 ± 0.31[b]	1.5 ± 0.27[b]	7.5 ± 1.13[a]	1.6 ± 0.33[b]	1.7 ± 0.28[b]
Dolichomastigales	0.0 ± 0.01[b]	0.1 ± 0.03[a]	0.2 ± 0.04[a]	0.0 ± 0.02[b]	0.2 ± 0.04[a]	0.2 ± 0.04[a]
Mamiellales	7.9 ± 1.13[a]	1.8 ± 0.29[b]	1.3 ± 0.27[b]	7.4 ± 1.14[a]	1.4 ± 0.33[b]	1.6 ± 0.28[b]
Bathycoccaceae	4.3 ± 0.64[a]	1.7 ± 0.28[b]	1.1 ± 0.26[b]	4.1 ± 0.63[a]	1.3 ± 0.30[b]	1.5 ± 0.30[b]
Mamiellaceae	3.6 ± 0.59[a]	0.1 ± 0.04[b]	0.1 ± 0.03[b]	3.3 ± 0.60[a]	0.1 ± 0.03[b]	0.1 ± 0.05[b]
Prasino-Clade-I	0.0 ± 0.01	0.0 ± 0.00	0.0 ± 0.00	0.0 ± 0.01	0.0 ± 0.00	0.0 ± 0.00
Prasino-Clade-V	0.2 ± 0.05	0.4 ± 0.12	0.2 ± 0.1	0.2 ± 0.04	0.3 ± 0.13	0.2 ± 0.07
Prasinococcales	0.0 ± 0.01	0.0 ± 0.01	0.0 ± 0.02	0.0 ± 0.00[b]	0.0 ± 0.01[ab]	0.1 ± 0.02[a]
RHIZARIA	1.9 ± 0.55	1.5 ± 0.67	3.2 ± 1.03	1.8 ± 0.53	3.0 ± 1.01	2.0 ± 0.89
Cercozoa	1.9 ± 0.55	1.5 ± 0.67	3.2 ± 1.03	1.8 ± 0.53	3.0 ± 1.01	2.0 ± 0.89
Filosa-Chlorarachnea	0.1 ± 0.03	0.0 ± 0.01	0.0 ± 0.00	0.1 ± 0.03	0.0 ± 0.01	0.0 ± 0.00
Filosa-Thecofilosea	1.8 ± 0.55	1.4 ± 0.66	3.2 ± 1.03	1.7 ± 0.52	3.0 ± 1.01	2.0 ± 0.89
AMOEBOZOA	0.1 ± 0.04	0.1 ± 0.04	0.0 ± 0.02	0.1 ± 0.04	0.0 ± 0.01	0.1 ± 0.05
APUSOZOA	0.1 ± 0.03	0.2 ± 0.14	0.0 ± 0.01	0.1 ± 0.03	0.0 ± 0.01	0.2 ± 0.16
Apusomonadidae	0.1 ± 0.03	0.0 ± 0.01	0.0 ± 0.00	0.1 ± 0.03	0.0 ± 0.00	0.0 ± 0.01

Significant differences ($P \leq 0.05$) were indicated with different superscript letters.
